# Efficient Removal of Butachlor and Change in Microbial Community Structure in Single-Chamber Microbial Fuel Cells

**DOI:** 10.3390/ijerph16203897

**Published:** 2019-10-15

**Authors:** Xiaojing Li, Yue Li, Lixia Zhao, Yang Sun, Xiaolin Zhang, Xiaodong Chen, Liping Weng, Yongtao Li

**Affiliations:** 1 Agro-Environmental Protection Institute, Ministry of Agriculture and Rural Affairs/Key Laboratory of Original Agro-Environmental Pollution Prevention and Control, MARA/Tianjin Key Laboratory of Agro-Environment and Agro-Product Safety, Tianjin 300191, China; lixiaojing@caas.cn (X.L.); lidabaoyy@163.com (Y.L.); zhaolixia@caas.cn (L.Z.); sunyang01@caas.cn (Y.S.); zhangxl826zxl@163.com (X.Z.); xiaodongchen1@sina.com (X.C.); liping.weng@wur.nl (L.W.); 2 College of Natural Resources and Environment, South China Agricultural University, Guangzhou 510642, China

**Keywords:** microbial electrochemical technology, herbicide-polluted wastewater, butachlor degradation, microbial community, dominant microbe change

## Abstract

Microbial electrochemical technology provides an inexhaustible supply of electron acceptors, allowing electroactive microorganisms to generate biocurrent and accelerate the removal of organics. The treatment of wastewater contaminated by butachlor, which is a commonly used chloroacetamide herbicide in paddy fields, is a problem in agricultural production. In this study, butachlor was found to be removed efficiently (90 ± 1%) and rapidly (one day) in constructed single-chamber microbial fuel cells (MFCs). After the addition of sodium acetate to MFCs with butachlor as the sole carbon source, electricity generation was recovered instead of increasing the degradation efficiency of butachlor. Meanwhile, the microbial community structure was changed in anodic and cathodic biofilms after the addition of butachlor, following the bioelectrochemical degradation of butachlor. High-throughput sequencing showed the proliferation of *Paracoccus* and *Geobacter* in MFCs with butachlor as the sole carbon source and of *Thauera butanivorans* in MFCs with butachlor and sodium acetate as concomitant carbon sources. These species possess the ability to oxidize different substituents of butachlor and have important potential use for the bioremediation of wastewater, sediments, and soils.

## 1. Introduction

Chloroacetamide herbicides, e.g., alachlor, acetochlor, butachlor, and metolachlor, are commonly used herbicides in rice, corn, soybean, and many other crops for controlling annual grass and broadleaf weeds [1,2]. Butachlor (*N*-(butoxymethyl)-2-chloro-*N*-(2,6-diethylphenyl) acetamide) is a widely used chloroacetamide herbicide in paddy fields. Exterior drainage and outdoor rainwater are inevitably polluted by residues of butachlor and its degradation metabolites, which pose a threat to the surrounding environment, especially ground and surface waters, since they are highly toxic to some aquatic organisms [3] and are potentially carcinogenic (e.g., butachlor caused stomach tumors in rats [4]). Therefore, this risky wastewater needs to be treated before discharge.

The biodegradation of organic pollutants was demonstrated as a feasible and safe treatment technology [1,5,6]. However, the degradation efficiency is seriously limited in anoxic environments, e.g., groundwater, sediment, and subsoil [7,8]. Moreover, butachlor and alachlor are slower to degrade than acetochlor and metolachlor, since it is possible that the electronic effect of the long-chain alkyl substituents alters the susceptibility of the benzene ring [9]. Presently, microbial electrochemical technology that provides an inexhaustible solid anode as the electron acceptor, e.g., microbial fuel cells (MFCs), can efficiently and rapidly degrade organic pollutants in anoxic environments and simultaneously generate electricity by means of electroactive microorganisms [10,11,12,13].

The objective of this study was to investigate the removal efficiency of butachlor in constructed single-chamber MFCs with and without an easily assimilated carbon source to elucidate whether there was a co-metabolism effect. Moreover, the capacity of electricity generation was demonstrated in MFCs using butachlor as the sole and concomitant carbon source. Finally, the change in microbial community structure was revealed in the anodic and cathodic biofilms of MFCs, especially with regard to predominant microbes, which possess potential use for the bioremediation of wastewaters, sediments, and soils.

## 2. Materials and Methods

### 2.1. MFC Construction and Operation

The MFCs used were cylinder reactors (4 cm in length and 3 cm in diameter) composed of an activated-carbon air cathode and a carbon-fiber brush anode as described in previous reports [14,15,16]. All MFCs were inoculated using the effluent of a mature MFC operated for over one year. The medium was a phosphate buffer nutrient solution (PBS) containing 10.317 g/L Na_2_HPO_4_·12H_2_O, 3.321 g/L NaH_2_PO_4_·2H_2_O, 0.31 g/L NH_4_Cl, 0.13 g/L KCl, 12.5 mL/L trace minerals, 5 mL/L vitamins, and 1.0 g/L sodium acetate (NaAC) [17,18]. The external resistance (*R*) was 1000 Ω, and the voltage output (*U*) was recorded by a data acquisition system (PISO-813U, ICP DAS Co., Ltd., Shanghai, China). The accumulated charge of one cycle was calculated as $Q=\;\int_{0}^{T}\frac{U}{R}\mathrm{dt}$, where t = 1800 s used to represent one unit. The medium was replaced when the voltage output was lower than 10 mV in a 30 °C incubator.

### 2.2. Addition of Butachlor and MFC Acclimation

MFCs were successfully started-up when the maximum voltage outputs were almost consistent over five successive cycles. Then, a concentration array including 10, 20, 40, 80, 160, 320, 640, 1000, 2000, and 4000 μg/L butachlor was added into the medium, with one concentration used for each cycle (one day). In the 4000 μg/L treatment, MFCs were steady for eight cycles. Subsequently, the NaAC was removed from the medium and 4000 μg/L butachlor was used as the sole carbon source (marked as BUT). MFCs with NaAC as the sole carbon source were used as the control for electricity generation and microbial community analysis, while the removal rate of butachlor in a sample bottle under the same conditions was used as the control for the non-MFC treatment. After nine cycles, the cathodic and anodic biofilms were extracted and marked as BUT.C and BUT.A, respectively. Then, the NaAC was again added to the MFCs with butachlor as the carbon source (marked as BUT-NaAC) and operated for another 11 cycles. These cathodic and anodic biofilms were also extracted and marked as BUT.C-NaAC and BUT.A-NaAC, respectively.

### 2.3. Measurement of Butachlor

The extraction procedure of butachlor was as follows: 10 mL of solution and 10 mL of acetonitrile were blended and vortexed for 5 min. Then, 5 g of NaCl was added and again vortexed for 5 min, followed by standing for layering of the blended solution. Subsequently, the mixture of supernatant (1.5 mL), Cleanert primary secondary amine sorbent (0.05 g, Agela Technologies), and anhydrous magnesium sulfate (0.15 g) was vortexed for 2 min and centrifuged at 4000 r/min for 2 min. Then, 1 mL of supernatant was dried by nitrogen blowing and diluted into 1 mL of *n*-hexane. The content of butachlor was quantified by a gas chromatograph (Agilent 7890B, Agilent Technologies Inc., Santa Clara, CA, USA) with an electron capture detector and an HP-5 column (30 m length × 0.32 mm inner diameter and 0.25 μm film thickness) for elution. The vaporizer and detector temperatures were 250 and 300 °C, respectively. Then, 2 μL of sample was injected and the column temperature protocol was as follows: 80 °C for 1 min, to 200 °C at a rate of 50 °C/min, and then 260 °C at a rate of 20 °C/min, before standing for 2 min. High-purity nitrogen was used as the carried gas at a flow rate of 3 mL/min, with a retention time of 7.33 min. Each measurement was repeated three times. The concentration value of butachlor was calculated as *C* = *C*_0_ − *C*_t_, where *C*_0_ and *C*_t_ are the initial concentration and the concentration after a one-cycle reaction.

### 2.4. Microbial Community Analysis

Genomic DNA was extracted using a commercial soil DNA extraction kit (Tiangen, Beijing, China), and the purity was confirmed using 1% agarose gel electrophoresis. The primers used were 515F (5′–GTGCCAGCMGCCGCGGTAA–3′) and 806R (5′–GGACTACHVGGGTWTCTAAT–3′) for identifying the bacterial diversity in the V4 region of 16S [19]. The PCR reactions were conducted in 30-μL reactions containing 15 μL of PCR Master (Thermo Scientific™, Waltham, MA, USA), 0.2 μL each of forward and reverse primers (15 μM), and 10 ng of template DNA. The PCR procedure was as follows: initial denaturation at 98 °C for 1 min, followed by 30 cycles of denaturation at 98 °C for 10 s, annealing at 50 °C for 30 s, and elongation at 72 °C for 30 s, and finally 72 °C for 5 min. After visualization by agarose gel electrophoresis (2%), the PCR products were purified using an AxyPrep DNA gel extraction kit (Qiagen, Hilden, Germany). The libraries of samples were established using a TruSeq^®^ DNA PCR-Free Sample Preparation kit (Illumina, San Diego, CA, USA), after sequencing on a HiSeq2500 PE250 by the specialized company Novogene (Beijing, China). Raw reads from paired-end sequencing were spliced to gain effective tags. Then, operational taxonomic units (OTUs) were clustered at a similarity of 97%, and the species annotation was conducted based on the Silva database. Finally, the Alpha diversity, based on the Chao 1, ACE (http://www.mothur.org/wiki/Ace), Shannon, and Simpson indices, was calculated using Qiime software (Version 1.7.0, QIIME development team, Boulder, CO, USA).

## 3. Results

### 3.1. Butachlor Removal and Electricity Generation

**Figure 1:**
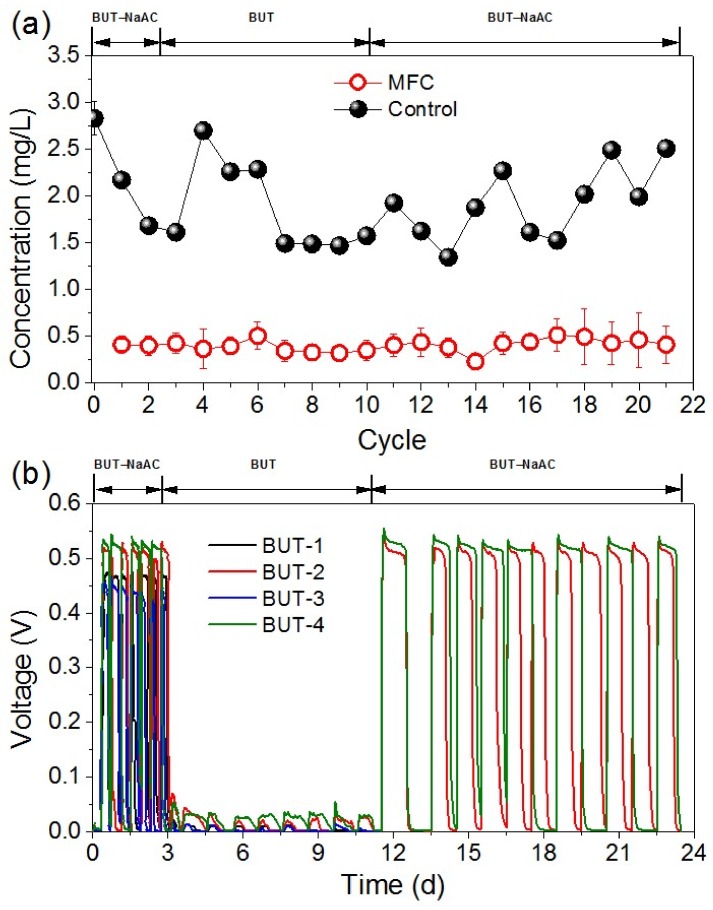
Butachlor concentration (**a**) and electricity production (**b**) in single-chamber microbial fuel cells (MFCs). Phase I: addition of butachlor and sodium acetate (BUT-NaAC), phase II: addition of only butachlor (BUT), and phase III: concomitant addition of butachlor and sodium acetate (BUT-NaAC). MFCs with NaAC as the sole carbon source were used as the control for electricity generation, while the removal rate of butachlor in a sample bottle under the same conditions was used as the control for non-MFC treatment. Bars denote the standard error. BUT-1, -2, -3, and -4 represent the four treatments with the addition of butachlor.

The removal efficiency of 4000 μg/L butachlor was maintained at 90 ± 1% in the eight-cycle treatment of BUT, while it was maintained at 89 ± 2% in the 11-cycle treatment of BUT-NaAC (Figure 1a). In contrast, 52–53% removal rates were obtained in the corresponding controls which were placed in the same conditions without MFC treatment. Additionally, the charge output in one cycle decreased from 325 ± 31 C (averaged in 22 cycles) in MFCs with sodium acetate and butachlor as the concomitant carbon sources to 11 ± 8 C (30 cycles) in BUT with butachlor as the sole carbon source (Figure 1b). Subsequently, the electricity generation was recovered to 349 ± 81 C (18 cycles) in BUT-NaAC with butachlor and sodium acetate as the concomitant carbon sources.

### 3.2. Microbial Community Richness and Diversity Indices

**Figure 2:**
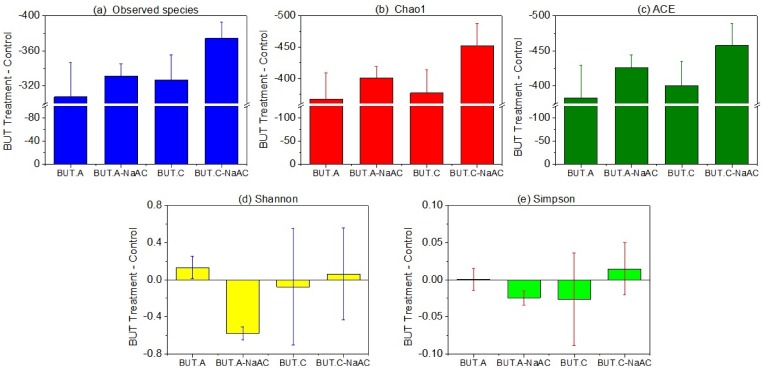
The Alpha diversity index changes on the anode (A) and cathode (C) of MFCs; (**a**): observed species, (**b**): Chao 1 index, (**c**): ACE index, (**d**): Shannon index and (**e**): Simpson index; *Y*-axis = BUT treatment−control. The values of controls are shown in Table S1 (Supplementary Materials). The data are presented as means ± standard error (SE) from duplicate experiments.

The microbial richness of anodic and cathodic biofilms obviously decreased in BUT and BUT-NaAC compared to the corresponding controls that used sodium acetate as the sole carbon source (Table S1, Supplementary Materials) based on the following indices: 42–49% less observed species, 45–52% lower Chao 1 index, and 46–52% lower ACE index (Figure 2a–c). Moreover, the addition of sodium acetate inhibited the richness of both anodic and cathodic biofilms to a certain extent (2–4% decrease) in BUT-NaAC relative to BUT. Compared to the controls, the microbial diversity of the anodic biofilm increased while that of the cathodic biofilm decreased (Figure 2d,e), with butachlor as the sole carbon source (in BUT). Subsequently, an opposite trend was observed after joint addition of butachlor and sodium acetate (in BUT-NaAC); for example, the Shannon index of the anodic biofilm in BUT-NaAC showed a 10 ± 1% decrement. 

### 3.3. Change in Microbial Abundances at the Class Level

**Figure 3:**
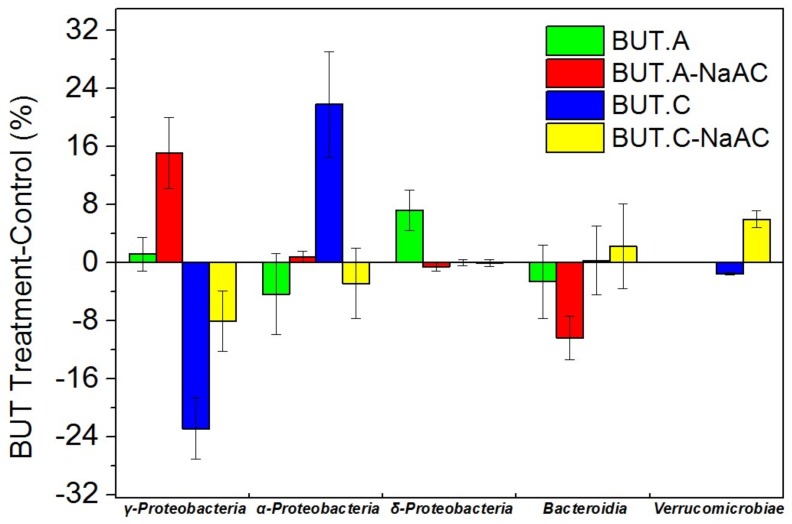
The top five microbial abundances at the class level on the anode (A) and cathode (C) of MFCs; *Y*-axis = BUT treatment−control. The values of controls are shown in Table S2 (Supplementary Materials). The data are presented as means ± SE from duplicate experiments. The total abundances of the top five classes accounted for 88–95% of the total community.

The total abundances of the top 11 classes accounted for 96–99% of all 58 identified classes in the anodic and cathodic biofilms (Table S2, Supplementary Materials). Therein, the abundance changes of the top five classes exhibited relatively obvious variation (6–23% relative abundance changes) compared to the corresponding controls and, thus, their changes were analyzed (Figure 3). Their abundances accounted for 88–94% and 88–95% of the anodic and cathodic microbial communities, respectively. Furthermore, Proteobacteria, containing α-Proteobacteria, γ-Proteobacteria, and δ-Proteobacteria, were dominant and their abundances accounted for 64–80% and 71–81% of the anodic and cathodic amounts, respectively. Compared to the controls, the abundance of γ-Proteobacteria increased by 15 ± 5%, while that of Bacteroidia decreased by 10 ± 3% in the anodic biofilm of BUT-NaAC. In the cathodic biofilm, the abundance of γ-Proteobacteria descended by 8 ± 4%, while that of Verrucomicrobiae increased by 6 ± 1%. Additionally, the amount of α-Proteobacteria increased by 22 ± 7%, whereas that of γ-Proteobacteria dropped by 23 ± 4% in the cathodic biofilm of BUT. In the anodic biofilm, the abundance of δ-Proteobacteria increased by 7 ± 3%, while those of α-Proteobacteria and Bacteroidia decreased by 3–4%.

### 3.4. Change in Microbial Abundances at the Genus Level

**Figure 4:**
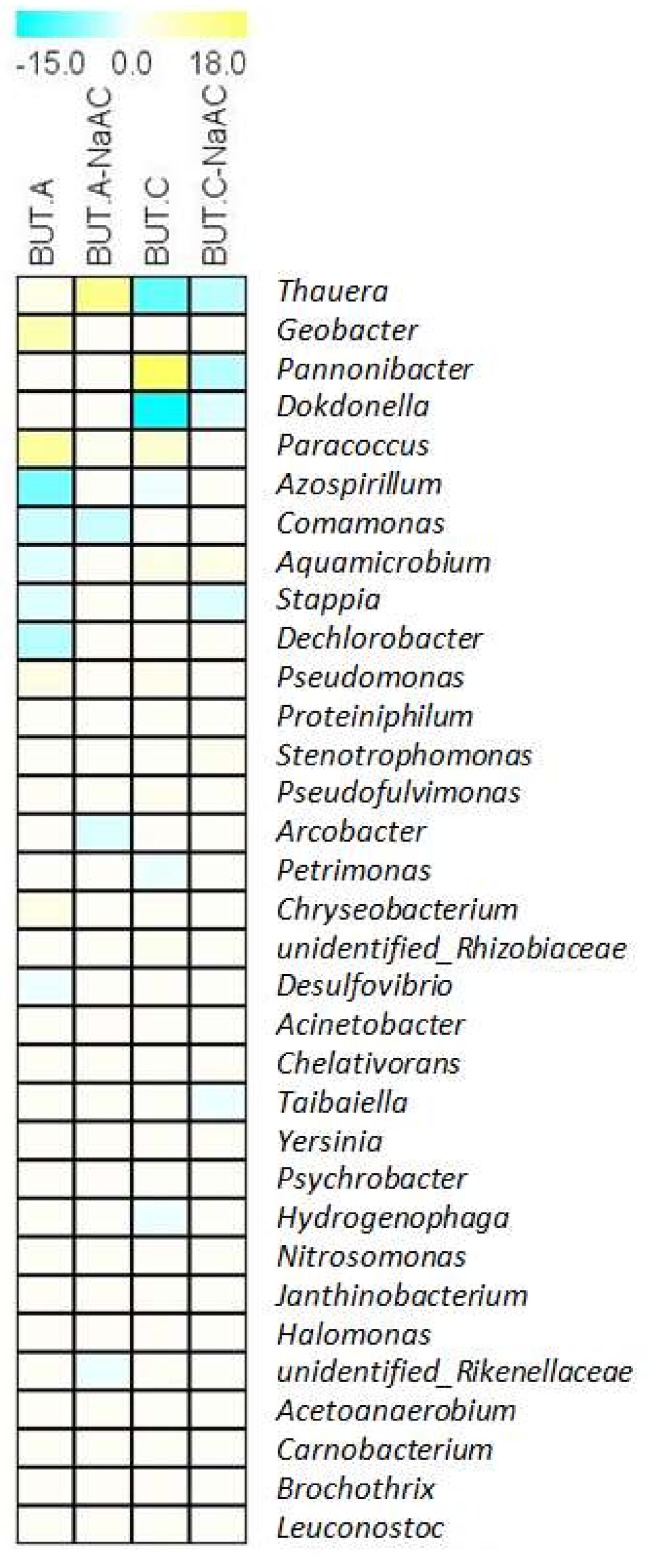
The 33 genera relative abundances (%) on the anode (A) and cathode (C) of MFCs; the values are represented in color as the difference between BUT treatment and control. Data are shown for when absolute values were greater than one. The values of controls are shown in Table S3 (Supplementary Materials).

The top 33 genera were analyzed in detail, since their abundance changes were greater than 1% (Figure 4). The abundance of *Thauera* (γ-Proteobacteria) was the highest and increased to 22 ± 4% in the anodic biofilm of BUT-NaAC from 9 ± 3% in the control, whereas it decreased by 4–9% in the cathodic biofilms with the addition of butachlor. Furthermore, *Thauera butanivorans* was predominant at the species level, and its abundance accounted for 22 ± 4% in the anodic biofilm of BUT-NaAC. In the cathodic biofilm of BUT, the abundance of *Pannonibacter* (mainly *Pannonibacter phragmitetus*) belonging to Rhizobiales (α-Proteobacteria) increased to 26 ± 13% from 8 ± 4% in the control. Additionally, in γ-Proteobacteria, *Dokdonella* (mainly *Dokdonella ginsengisoli*) belonging to Rhodanobacteraceae increased to 16 ± 9% from 1 ± 0.7%. Interestingly, the amount of *Geobacter* reached up to 16 ± 3% in the anodic biofilm of BUT, a 9% increment relative to the control, which was comparable to that (12 ± 1%) for BUT-NaAC. The abundance of *Paracoccus* belonging to Rhodobacteraceae (α-Proteobacteria) reached to 11 ± 6% in the anodic biofilm of BUT from a low amount in the control, and subsequently decreased to 3 ± 0.2% for BUT-NaAC. In the anodic and cathodic biofilms of BUT, the abundances of *Azospirillum* (α-Proteobacteria) decreased to 0.6 ± 0.2% and 1 ± 0.3% from 8 ± 6% and 6 ± 2% in the control, respectively.

## 4. Discussion

This study demonstrated that butachlor could be removed efficiently (90 ± 1%) and rapidly (one day) in activated-carbon air cathode MFCs, while the observed electricity generation was unsurprising. Unexpectedly, the abundance of *Geobacter* increased distinctly instead of decreasing in the anodic biofilm of MFCs with butachlor as the sole carbon source (in BUT). It was expected that electrons generated by the electroactive microorganisms would be consumed by the biodegradation of butachlor, but they were not. The charge output and removal efficiency of butachlor in MFCs with butachlor and sodium acetate as the concomitant carbon sources (in BUT-NaAC) were comparable to controls with sodium acetate as the sole carbon source and BUT, respectively. These results indicated that butachlor is unsuitable as fuel for MFCs; however, *Geobacter* may independently and/or synergistically metabolize the butachlor. For example, species of *Geobacter* have the ability to anaerobically oxidize aromatic compounds to benzoyl-coenzyme A, then to acetyl-coenzyme A via fatty-acid oxidation, and finally to carbon dioxide via the tricarboxylic acid cycle [20,21,22].

The amount of electricity generation in one cycle of BUT-NaAC was more than the totals of BUT and control alone. On the one hand, this suggests that some degradates (small molecular organics) were presumably transferred into electricity via the catalysis of microbes in the MFCs. On the other hand, the microbial richness in the anodic biofilm of BUT-NaAC apparently decreased compared to the control, while the abundance of *Geobacter* slightly increased. This suggests that there were fewer microbes to compete for the substrates with electroactive microorganisms, thereby generating more electricity. This study shows that it is infeasible to use sodium acetate as a co-metabolized carbon source to degrade butachlor. In fact, the concomitant use of sodium acetate possibly suppressed the activity of butachlor degradation, since lower microbial richness and diversity were found in the anodic biofilm of BUT-NaAC than in that of BUT. Therefore, a slightly low removal rate of butachlor was observed with BUT-NaAC.

In treatments with the addition of butachlor (BUT and BUT-NaAC), the microbial richness and diversity of biofilms showed an obvious change. In BUT, the abundances of *Paracoccus* and *Geobacter* significantly increased in the anodic biofilm compared to the control, suggesting a potential decomposition effect. Previous studies found that *Paracoccus* spp. could efficiently degrade chloroacetamide herbicides, e.g., butachlor, alachlor, and acetochlor [6]. Some species of *Paracoccus* could completely mineralize chlorpyrifos, in addition to degrading 3,5,6-trichloro-2-pyridinol, pyridine, methyl parathion, and carbonfuran [5]. Species from *Geobacter* mainly decomposed phenol [20], benzene [22], and benzoate [21] in anaerobic conditions. Thus, it can be inferred that species of *Paracoccus* possess good dechlorination/degradation ability, while species of *Geobacter* are able to oxidize phenyl alkyl substituents. In BUT-NaAC, the amount of *T. butanivorans* obviously increased in the anodic biofilm compared to the control. *T. butanivorans* is a C2–C9 alkane-oxidizing bacterium [23] and was, thus, deemed to degrade the butachlor. Moreover, this bacterium has the ability to secrete soluble butane monooxygenase [24] and, therefore, was able to oxidize the alkoxybutyl substituent, which is the limiting step of degradation efficiency for chloroacetamide herbicides [6]. Furthermore, the majority of species of *Thauera* have denitrification ability [25], which further stimulates the decomposition of butachlor, involving the amide nitrogen’s alkoxybutyl, which significantly affects the biodegradability of these herbicides [6]. Unfortunately, the degradation product analysis of butachlor was unsuccessful in this study, presumably due to the low aqueous solubility of degradates [9], which will be addressed in future work to reveal the bioelectrochemical degradation pathway and to assess the function of these special microbes.

## 5. Conclusions

Firstly, butachlor, a commonly used chloroacetamide herbicide in paddy fields, could be removed efficiently and rapidly in constructed single-chamber MFCs. The concomitant addition of sodium acetate recovered the electricity generation instead of increasing the degradation efficiency of butachlor. Secondly, a change in microbial community structure was induced after the addition of butachlor, in order to adapt to the bioelectrochemical degradation of butachlor. *Paracoccus* and *Geobacter* in BUT and *T. butanivorans* in BUT-NaAC proliferated, presumably following the oxidization of different substituents of butachlor; thus, they have important potential use for the bioremediation of wastewater, sediments, and soils.

## Supplementary Materials

The following are available online at https://www.mdpi.com/1660-4601/16/20/3897/s1, Table S1: The Alpha-diversity indices of controls. The CK and CK-NaAC were the control for BUT and BUT-NaAC, respectively. The value was mean ± SE with two duplicates, Table S2: The abundance changes of BUT treatment−Control. The CK and CK-NaAC were the controls of BUT and BUT-NaAC, respectively. The value was mean ± SE with two duplicates, Table S3: The microbial abundance of controls at the genus level. The CK and CK-NaAC were the controls of BUT and BUT-NaAC, respectively.

## References

[1] Fenner K., Canonica S., Wackett L.P., Elsner M. (2013). Evaluating pesticide degradation in the environment: Blind spots and emerging opportunities. Science.

[2] Woodward E.E., Hladik M.L., Kolpin D.W. (2018). Occurrence of dichloroacetamide herbicide safeners and co-applied herbicides in midwestern U.S. streams. Environ. Sci. Technol. Lett..

[3] Mccarroll N.E., Stack H.F.F., Protzel A., Ioannou Y., Jackson M.A., Waters M.D., Dearfield K.L. (2002). A survey of EPA/OPP and open literature on selected pesticide chemicals: III. Mutagenicity and carcinogenicity of benomyl and carbendazim. Mutat Res..

[4] Coleman S., Linderman R., Hodgson E., Rose R.L. (2000). Comparative metabolism of chloroacetamide herbicides and selected metabolites in human and rat liver microsomes. Environ. Health Persp..

[5] Xu G.M., Zheng Y.Y., Wang S.H., Zhang J.S., Yan Y.C. (2008). Biodegradation of chlorpyrifos and 3,5,6-trichloro-2-pyridinol by a newly isolated *Paracoccus* sp. strain TRP. Int. Biodeter. Biodegr..

[6] Zhang J., Zheng J.W., Liang B., Wang C.H., Cai S., Ni Y.Y., He J., Li S.P. (2011). Biodegradation of chloroacetamide herbicides by *Paracoccus* sp. FLY-8 in vitro. J. Agric. Food Chem..

[7] Accinelli C., Dinelli G., Vicari A., Catizone P. (2001). Atrazine and metolachlor degradation in subsoils. Biol. Fert. Soils.

[8] Bedmar F., Gimenez D., Costa J.L., Daniel P.E. (2017). Persistence of acetochlor, atrazine and *S*-metolachlor in surface and sub-surface horizons of two typic argiudolls under no-tillage. Environ. Toxicol. Chem..

[9] Friedman C.L., And A.T.L., Hay A. (2006). Degradation of chloroacetanilide herbicides by anodic fenton treatment. J. Agric. Food Chem..

[10] Li W., Yu H., Rittmann B.E. (2015). Chemistry: Reuse water pollutants. Nature.

[11] Logan B.E., Rabaey K. (2012). Conversion of wastes into bioelectricity and chemicals by using microbial electrochemical technologies. Science.

[12] Wang H., Luo H., Fallgren P.H., Jin S., Ren Z.J. (2015). Bioelectrochemical system platform for sustainable environmental remediation and energy generation. Biotechnol. Adv..

[13] Zhao F., Slade R.C.T., Varcoe J.R. (2009). Techniques for the study and development of microbial fuel cells: An electrochemical perspective. Chem. Soc. Rev..

[14] Li X., Wang X., Zhang Y., Ding N., Zhou Q. (2014). Opening size optimization of metal matrix in rolling-pressed activated carbon air–cathode for microbial fuel cells. Appl. Energ..

[15] Li X., Wang X., Zhang Y., Gao N., Li D., Zhou Q. (2015). Effects of catalyst layer and gas diffusion layer thickness on the performance of activated carbon air-cathode for microbial fuel cells. Int. J. Electrochem. Sci..

[16] Zhang Y., Wang X., Li X., Gao N., Wan L., Feng C., Zhou Q. (2014). A novel and high performance activated carbon air-cathode with decreased volume density and catalyst layer invasion for microbial fuel cells. RSC Adv..

[17] Lovley D.R., Phillips E.J. (1988). Novel mode of microbial energy metabolism: Organic carbon oxidation coupled to dissimilatory reduction of iron or manganese. Appl. Environ. Microbiol..

[18] Wan Y., Zhou L., Wang S., Liao C., Li N., Liu W., Wang X. (2018). Syntrophic growth of *Geobacter sulfurreducens* accelerates anaerobic denitrification. Front. Microbiol..

[19] Caporaso J.G., Lauber C.L., Walters W.A., Berg-Lyons D., Lozupone C.A., Turnbaugh P.J., Fierer N., Knight R. (2011). Global patterns of 16S rRNA diversity at a depth of millions of sequences per sample. Proc. Natl. Acad. Sci. USA.

[20] Schleinitz K.M., Schmeling S., Jehmlich N., Bergen M.V., Harms H., Kleinsteuber S., Vogt C., Fuchs G. (2009). Phenol degradation in the strictly anaerobic iron-reducing bacterium *Geobacter metallireducens* GS-15. Appl. Environ. Microb..

[21] Wischgoll S., Heintz D., Peters F., Erxleben A., Sarnighausen E., Reski R., Dorsselaer A.V., Boll M. (2005). Gene clusters involved in anaerobic benzoate degradation of *Geobacter metallireducens*. Mol. Microbiol..

[22] Zhang T., Tremblay P.L., Chaurasia A.K., Smith J.A., Bain T.S., Lovley D.R. (2013). Anaerobic benzene oxidation via phenol in *Geobacter metallireducens*. Appl. Environ. Microb..

[23] Dubbels B.L., Sayavedra-Soto L.A., Bottomley P.J., Arp D.J. (2009). *Thauera butanivorans* sp. nov., a C2-C9 alkane-oxidizing bacterium previously referred to as ‘*Pseudomonas butanovora*’. Int. J. Syst. Evol. Microbiol..

[24] Cooley R.B., Dubbels B.L., Sayavedra-Soto L.A., Bottomley P.J., Arp D.J. (2009). Kinetic characterization of the soluble butane monooxygenase from *Thauera butanivorans*, formerly ‘*Pseudomonas butanovora*’. Microbiology.

[25] Mao Y., Xia Y., Zhang T. (2013). Characterization of *Thauera*-dominated hydrogen-oxidizing autotrophic denitrifying microbial communities by using high-throughput sequencing. Bioresour. Technol..

