# Supplementary material for: Efficient Removal of Butachlor and Change in Microbial Community Structure in Single-Chamber Microbial Fuel Cells
Source: Int J Environ Res Public Health. 2019 Oct 15;16(20):3897. doi: 10.3390/ijerph16203897 (PMC6843377; doi:10.3390/ijerph16203897)
Supplement: Supplementary file 1 [file ijerph-16-03897-s001.pdf]

Supporting Information

Efficient Removal of Butachlor and Change in Microbial Community Structure in Single-Chamber Microbial Fuel Cells

Xiaojing Li [1], Yue Li [1], Lixia Zhao [1], Yang Sun [1], Xiaolin Zhang [1], Xiaodong Chen [1], Liping Weng [1] and Yongtao Li [1,2,*]

[1] Agro-Environmental Protection Institute, Ministry of Agriculture and Rural Affairs/Key Laboratory of Original Agro-Environmental Pollution Prevention and Control, MARA/Tianjin Key Laboratory of Agro-Environment and Agro-Product Safety, Tianjin 300191, China; lixiaojing@caas.cn (X.L.); lidabaoyy@163.com (Y.L.); zhaolixia@caas.cn (L.Z.); sunyang01@caas.cn (Y.S.); zhangxl826zxl@163.com (X.Z.); xiaodongchen1@sina.com (X.C.); liping.weng@wur.nl (L.W.)

[2] College of Natural Resources and Environment, South China Agricultural University, Guangzhou 510642, China

* Correspondence: yongtao@scau.edu.cn; Tel.: +86-22-2361-1021; Fax: +86-22-2361-3820

Received: 27 September 2019; Accepted: 12 October 2019; Published: 15 October 2019

Table S1. The Alpha-diversity indices of controls. The CK and CK-NaAC were the control for BUT and BUT-NaAC, respectively. The value was mean ± SE with two duplicates.

Controls	Observed Species	SE	Chao1	SE	ACE	SE	Shannon	SE	Simpson	SE
CK.A	736	347	816.2635	406.6385	840.496	426.817	4.876	0.382	0.9045	0.0035
CK.A-NaAC	720	267	820.0465	327.5175	845.4995	347.0835	5.612	0.087	0.938	0.002
CK.C	706.5	319.5	779.831	370.307	807.6485	397.9135	5.1765	0.3695	0.921	0.004
CK.C-NaAC	765	311	868.974	383.367	872.647	381.519	5.3305	0.1425	0.9145	5.00×10^{-4}

Table S2. The abundance changes of BUT treatment–Control. The CK and CK-NaAC were the controls of BUT and BUT-NaAC, respectively. The value was mean ± SE with two duplicates.

**Taxonomy**	**CK.A**	**CK.C**	**CK.A-NaAC**	**CK.C-NaAC**	**BUT.A**	**SE**	**BUT.A-NaAC**	**SE**	**BUT.C**	**SE**	**BUT.C-NaAC**	**SE**
γ-Proteobacteria	25.5	44.7	37.0	52.4	1.2	2.3	15.1	4.9	−22.9	4.2	−8.1	4.2
α–Proteobacteria	28.6	34.4	12.5	26.8	−4.3	5.6	0.8	0.8	21.8	7.3	−2.9	4.9
δ–Proteobacteria	10.3	2.4	14.8	2.8	7.2	2.8	−0.6	0.6	0.0	0.4	−0.1	0.5
Bacteroidia	27.8	11.2	23.5	7.8	−2.6	5.1	−10.3	3.0	0.3	4.7	2.2	5.8
Verrucomicrobiae	0.2	2.0	0.2	1.4	0.1	0.0	0.0	0.0	−1.5	0.1	6.0	1.2
unidentified_Bacteria	0.2	0.1	3.0	0.2	−0.1	0.0	−1.7	0.5	0.0	0.0	0.0	0.0
Spirochaetia	0.4	0.3	0.7	0.9	−0.1	0.0	−0.1	0.0	0.1	0.0	1.6	0.3
Clostridia	1.5	1.2	1.4	2.6	−0.2	0.0	−0.3	0.1	0.2	0.0	−0.4	2.0
Bacilli	0.7	0.7	1.0	0.9	0.4	0.4	0.9	0.0	1.5	0.3	0.6	0.1
Synergistia	1.9	0.3	1.4	0.3	−0.6	0.5	−0.8	1.4	−0.1	1.1	0.0	0.1
Actinobacteria	0.3	0.5	0.4	0.5	0.8	0.0	−0.1	0.0	0.8	0.2	0.0	0.0

Table S3. The microbial abundance of controls at the genus level. The CK and CK-NaAC were the controls of BUT and BUT-NaAC, respectively.

Taxonomy	CK.A	CK.C	CK.A.Na	CK.C.Na
Thauera	0.08	0.14	0.09	0.22
Geobacter	0.070923	0.008567	0.117681	0.013447
Pannonibacter	0.016669	0.078306	0.014998	0.074921
Dokdonella	0.007883	0.161963	0.007658	0.043021
Paracoccus	0.002144	0.005317	0.004449	0.006057
Azospirillum	0.08229	0.009844	0.009216	0.003857
Comamonas	0.039474	0.00421	0.081627	0.007214
Aquamicrobium	0.051637	0.050636	0.025998	0.032599
Stappia	0.027571	0.030709	0.006036	0.058682
Dechlorobacter	0.048253	0.001742	0.021091	0.002454
Pseudomonas	0.002299	0.002658	0.015069	0.027606
Proteiniphilum	0.007531	0.019088	0.01093	0.01057
Stenotrophomonas	0.000994	0.006078	0.008257	0.011162
Pseudofulvimonas	0.004739	0.019829	0.007869	0.023636
Arcobacter	0.001869	0.000606	0.027472	0.001417
Petrimonas	0.001699	0.006212	0.001262	0.001128
Chryseobacterium	0.003307	0.001163	0.01057	0.00294
unidentified_Rhizobiaceae	0.004055	0.009759	0.005465	0.012946
Desulfovibrio	0.017762	0.005817	0.007348	0.006783
Acinetobacter	0.000141	0.000148	0.011451	0.004943
Chelativorans	0.000212	0.00098	0.000261	0.000677
Taibaiella	0.001396	0.005345	0.002447	0.011804
Yersinia	0	0	0.003011	0.005733
Psychrobacter	0	0	0.000134	0.000035
Hydrogenophaga	0.000381	0.010471	0.000275	0.000282
Nitrosomonas	0.002172	0.002066	0.009682	0.002313
Janthinobacterium	0.000028	0.000402	0.002531	0.002644
Halomonas	0.000134	0.000303	0.00019	0.000346
unidentified_Rikenellaceae	0.001389	0.002066	0.010979	0.001319
Acetoanaerobium	0.000423	0.000642	0.001587	0.008765
Carnobacterium	0	0	0.000536	0.001135
Brochothrix	0	0	0.000106	0.000085
Leuconostoc	0	0	0.000028	0.000042
